# An intellectual disability syndrome with single-nucleotide variants in O-GlcNAc transferase

**DOI:** 10.1038/s41431-020-0589-9

**Published:** 2020-02-20

**Authors:** Veronica M. Pravata, Michaela Omelková, Marios P. Stavridis, Chelsea M. Desbiens, Hannah M. Stephen, Dirk J. Lefeber, Jozef Gecz, Mehmet Gundogdu, Katrin Õunap, Shelagh Joss, Charles E. Schwartz, Lance Wells, Daan M. F. van Aalten

**Affiliations:** 1 Division of Gene Regulation and Expression and School of Life Sciences, University of Dundee, Dundee, UK; 2 Division of Cell and Developmental Biology, School of Life Sciences, University of Dundee, Dundee, UK; 3 Department of Biochemistry and Molecular Biology and Chemistry, Complex Carbohydrate Research Center, University of Georgia, Athens, GA USA; 4 Department of Neurology, Donders Institute for Brain, Cognition and Behaviour, Radboud University Medical Centre, 6500 HB Nijmegen, The Netherlands; 5 Adelaide Medical School and the Robinson Research Institute, The University of Adelaide, Adelaide, SA Australia; 6 Institute of Molecular Cell and System Biology, University of Glasgow, Glasgow, UK; 7 Department of Clinical Genetics, United Laboratories, Tartu University Hospital, Tartu, Estonia; 8 Department of Clinical Genetics, Institute of Clinical Medicine, University of Tartu, Tartu, Estonia; 9 West of Scotland Genetic Service, Queen Elizabeth University Hospital, Glasgow, UK; 10 Greenwood Genetic Center, Greenwood, SC 29646 USA; 11 Institute of Molecular Precision Medicine, Xiangya Hospital, Central South University, Changsha, China

**Keywords:** Development, Neurodevelopmental disorders

## Abstract

Intellectual disability (ID) is a neurodevelopmental condition that affects ~1% of the world population. In total 5−10% of ID cases are due to variants in genes located on the X chromosome. Recently, variants in *OGT* have been shown to co-segregate with X-linked intellectual disability (XLID) in multiple families. *OGT* encodes O-GlcNAc transferase (OGT), an essential enzyme that catalyses O-linked glycosylation with β-N-acetylglucosamine (O-GlcNAc) on serine/threonine residues of thousands of nuclear and cytosolic proteins. In this review, we compile the work from the last few years that clearly delineates a new syndromic form of ID, which we propose to classify as a novel Congenital Disorder of Glycosylation (OGT-CDG). We discuss potential hypotheses for the underpinning molecular mechanism(s) that provide impetus for future research studies geared towards informed interventions.

## Introduction

Intellectual disability (ID) is an early-onset neurodevelopmental condition characterised by deficits in intelligence (IQ < 70) and concomitant defects in adaptive behaviour [1, 2]. An estimated 0.5−3% of the population in the developed world is affected by the condition [3–6]. Although ID can occur in isolation (nonsyndromic ID), it is often accompanied by a broad spectrum of other mental or physical limitations (syndromic ID). Causes underlying ID are heterogeneous [7–9]; and the aetiology of ~30% of ID cases is unknown [9]. Monogenic causes account for 40% of all ID with a genetic component, yet, one of over 800 genes can be involved. Since X-linked genes were shown to be expressed more abundantly in the brain than in any other tissue [10], the X chromosome has a disproportionately higher number of genes implicated in mental ability compared with other chromosomes [11, 12]. Indeed, aberrations in at least 140 genes located on the X chromosome were found to cause X-linked intellectual disability (XLID) [13–16], although several candidate genes remain controversial [13].

Human O-GlcNAc transferase (*OGT*), located on the X chromosome (Xq13.1), encodes a 110 kDa protein [17, 18] that is highly conserved from *Caenorhabditis elegans* to *Homo sapiens* [19]. OGT catalyses O-linked glycosylation of nuclear, cytosolic, and mitochondrial proteins with β-N-acetylglucosamine (O-GlcNAc), which is an essential protein serine/threonine modification in vertebrata [19–21]. Attachment and removal of the O-GlcNAc moiety on mammalian nuclear and cytoplasmic proteins is performed by only two enzymes: OGT and O-GlcNAcase (OGA), respectively (Fig. 3). O-GlcNAcylation is thought to be involved in key cellular processes such as gene regulation and expression [22–24], metabolic activity [25], and cell-cycle regulation [26]. Changes in O-GlcNAc homoeostasis have been linked to severe developmental problems and neurodegenerative diseases [27–33].

**Fig. 1:**
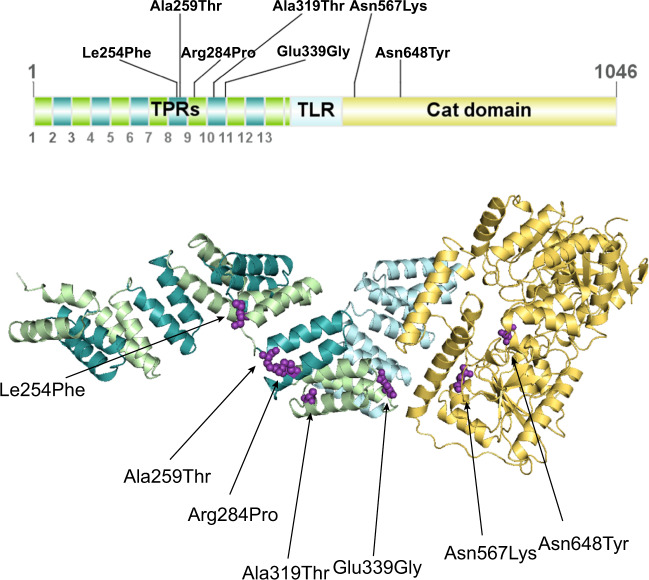
3D crystal structure of human OGT with mapped variants. Model for the full-length human OGT produced by superposition of crystallographic models for the human OGT catalytic core (Protein Data Bank code 5C1D) and TPR domain (Protein Data Bank code 1W3B). Green region represents the TPR, yellow region represents the CD, and purple highlighted residues represent variants found in OGT-XLID patients.

OGT is a multi-domain protein characterised by a catalytic domain (CD) at the C-terminus and an N-terminal tetratricopeptide repeat domain (TPR) that is involved in substrate recognition and protein–protein interactions (Fig. 1) [34–36]. OGT is essential for mouse embryonic stem cell (mESC) and somatic cell survival [19, 37], whereas ablation of *OGT* is embryonic lethal in mice [19], zebrafish [38], and *Drosophila* [39]. *Sxc*, the gene encoding *Drosophila* OGT (*Dm*OGT) belongs to the family of homeotic genes, the Polycomb group, which regulate segmentation during development [39–41]. *Sxc* loss of function leads to a super sex combs phenotype in *Drosophila* [39] and death in the adult pharate stage. Interestingly, in addition to its catalytic function, OGT promotes the proteolytic processing and activation of a chromatin-bound transcriptional co-regulator host cell factor 1 (HCF1) [42, 43].

**Fig. 2:**
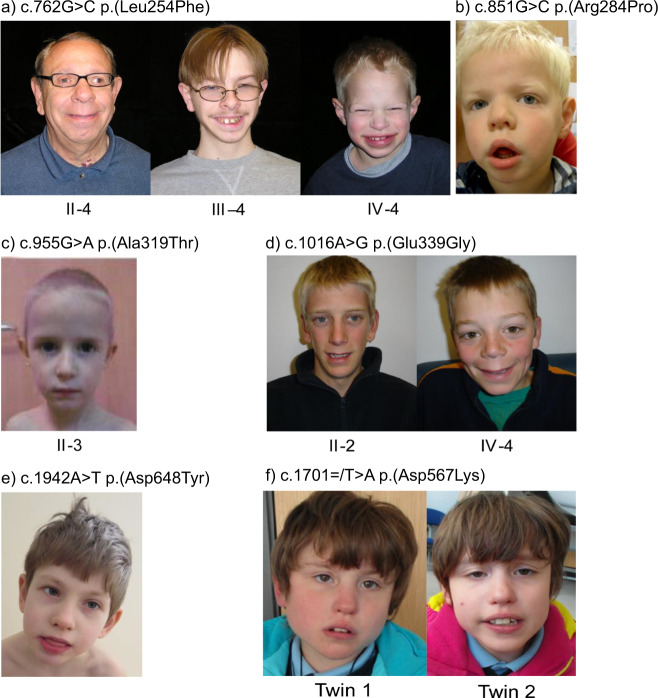
Facial photographs of patients with variants in OGT. **a** Three affected males from a family with c.762G>C p.(Leu254Phe) variant [30]. **b** One affected male with c.851G>C p.(Arg284Pro) variant [29]. **c** One affected male with c.955G>A p.(Ala319Thr) variant [44]. **d** Two affected males in family with c.1016A>G p.(Glu339Gly) variant [31]. **e** Affected male with c.1942A>T p.(Asn648Tyr) variant [33]. **f** Affected female twins with c.1701=/T>A p.(Asn567Lys) OGT variant [32]. Note the wide mouth, thin upper lip, full lower lip, and smooth philtrum in most of the males. The female twins have a full lower lip and twin 2 has a wide mouth.

Over the past 4 years, six reports have described the discovery of the first *OGT* variants causal for XLID [29–33, 44, 45] (OGT-XLID variants), suggesting a possible link between the O-GlcNAc system and neurodevelopment. Here, we first present common clinical features of these patients suggestive of a syndromic form of XLID (Fig. 2 and Table 1). We then discuss potential, and not mutually exclusive, hypotheses that could explain the cellular mechanisms underpinning neuropathogenesis (Fig. 1).

**Table 1:** Clinical findings observed in OGT-XLID.

OGT variant (reference sequence NM_181672.2)	c.762G>C p. (Leu254Phe)	c.775G>A p. (Ala259Thr)	c.851G>C p. (Arg284Pro)	c.955G>A p. (Ala319Thr)	c.1016A>G p. (Glu339Gly)	c.1942A>T p. (Asn648Tyr)	c.1701/T>A p. (Asn567Lys)	Total
	TPR domain variants					Catalytic domain variants		
Reference	Vaidyanathan et al. [30]	Selvan et al. [31]	Willems et al. [29]	Bouazzi et al. [44]	Selvan et al. [31]	Pravata et al. [32]	Pravata et al. [33]	
Number of individuals	*3 males*	*1 male*	*1 male*	*3 males*	*2 males*	*1 male*	2 females	13
Behavioural problems	*0/3*	*1/1*	*1/1*	*2/3*	*2/2*	*1/1*	NA	7/11
Hypotonia	*NA*	*NA*	*NA*	*NA*	*1/2*	*1/1*	2/2	**4/5**
Drooling	*NA*	*NA*	*NA*	*NA*	*NA*	*1/1*	NA	1/13
Genital/reproductive abnormalities	*2/3*	*0/1*	*1/1*	*NA*	*0/2*	*0/1*	NA	3/8
Epilepsy/seizures/dystonia	*0/3*	*1/1*	*NA*	*0/3*	*0/2*	*0/1*	NA	1/10
Eye abnormalities	*3/3*	*1/1*	*1/1*	*2/3*	*0/2*	*1/1*	2/2	**10/13**
Ear abnormalities/hearing impairment	*0/3*	*1/1*	*0/1*	*1/3*	*2/2*	*1/1*	NA	5/11
Brain abnormalities	*NA*	*1/1*	*1/1*	*0/3*	*0/2*	*1/1*	2/2	5/10
Microcephaly	*1/3*	*1/1*	*1/1*	*0/3*	*0/2*	*0/1*	0/2	3/13
Thin corpus callosum	*NA*	*1/1*	*NA*	*0/3*	*NA*	*0/1*	NA	1/5
Dysmorphic features	*3/3*	*1/1*	*1/1*	*2/3*	*2/2*	*1/1*	2/2	**12/13**
Dolichocephalic head	*NA*	*NA*	*NA*	*2/3*	*2/2*	*0/1*	NA	**4/6**
Frontal hair upsweep	*2/3*	*NA*	*NA*	*2/3*	*0/2*	*0/1*	NA	4/9
Full lips	*1/3*	*NA*	*1/1*	*NA*	*0/2*	*1/1*	NA	3/7
Full/long philtrum	*1/3*	*NA*	*NA*	*2/3*	*2/2*	*0/1*	NA	5/9
Broad nasal root	*NA*	*NA*	*1/1*	*NA*	*1/2*	*1/1*	NA	3/4
Clinodactyly	*3/3*	*0/1*	*1/1*	*1/3*	*1/2*	*Syndactyly*	2/2	9/13
Long thin fingers	*NA*	*NA*	*1/1*	*3/3*	*0/2*	*1/1*	NA	**5/7**
Developmental delay	*3/3*	*1/1*	*1/1*	*3/3*	*2/2*	*1/1*	2/2	**13/13**
Low birth weight	*NA*	*1/1*	*NA*	*2/3*	*1/2*	*0/1*	2/2	6/9
Short stature	*3/3*	*1/1*	*NA*	*2/3*	*0/2*	*0/1*	NA	6/10
Language delay/problems	*NA*	*NA*	*NA*	*3/3*	*2/2*	*1/1*	2/2	**8/8**
IQ	IQ 49, IQ 61, IQ 58	moderate ID	WPPSI-III-NL, score 2;9	IQ 40, IQ 35, IQ 30	Mild-to-moderate ID	Moderate-to-severe ID	ID	**13/13**

NA indicates cases where no information was available. Numbers indicate number of affected patients over number of patients examined per variant and for the phenotype. Information relating to male patients is highlighted in italics. Information relating to female patients is underlined. Frequently observed phenotypes are highlighted in bold.

### Clinical features of patients with OGT-XLID variants

**Fig. 3:**
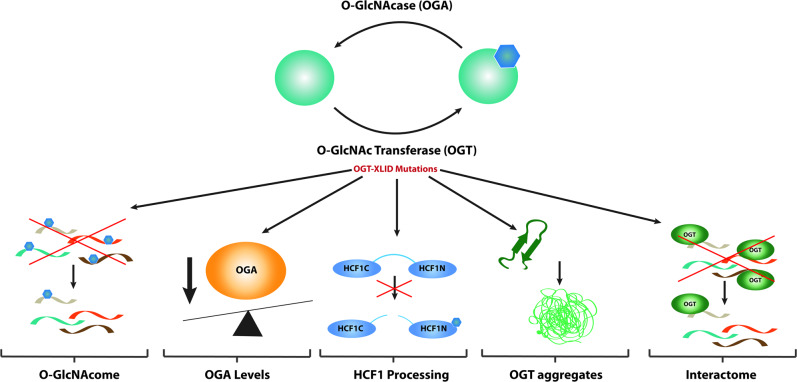
OGT may mediate XLID pathology via alterations in the O-GlcNAcome, HCF1 processing, or misfolding. OGT catalyses the transfer of O-GlcNAc moiety onto Ser/Thr residues of acceptor substrates. This modification is removed by the OGA enzyme. Variants in the OGT gene which lead to amino acid substitutions in the OGT protein may mediate the XLID pathophysiology via (1) downstream effect on the O-GlcNAc proteome, (2) alterations in OGA levels, (3) incorrect processing of the HCF1 transcriptional coregulatory HCF1 which is also encoded by an XLID-associated gene (HCFC1), (4) misfolding of OGT possibly leading to misfolded OGT aggregation, and (5) perturbation in OGT interactome.

Thirteen affected individuals from seven families presenting with previously unclassified ID and developmental delay were subjected to genetic testing and found to carry nonsynonymous *variants* in the *OGT* gene (NM_181672.2, GenBank) located on the X chromosome (Table 1). Three patients carried de novo variants resulting in single-amino acid variants in the CD of *OGT*, one patient with NM_181672.2:c.1942A>T p.(Asn648Tyr) [33, 46] and two patients with NM_181672.2:c.1701=/T>A p.(Asn567Lys) [32, 47], while all others carried inherited variants in the TPR domain of OGT NM_181672.2:c.762G>C p.(Leu254Phe) [30], NM_181672.2:c.775G>A p.(Ala259Thr) [31], NM_181672.2:c.851G>C p.(Arg284Pro) [29], NM_181672.2:c.955G>A p.(Ala319Thr) [44], NM_181672.2:c.1016A>G p.(Glu339Gly) [31], (Fig. 3). Four of the identified variants are found in multiple probands from the same families: three males with the c.762G>C p.(Leu254Phe) variant, three brothers with the c.955G>A p.(Ala319Thr) variant, two brothers with the c.1016A>G p.(Glu339Gly) variant, female twins with the c.1701=/T>A p.(Asn567Lys) variant and a male with c.1942A>T p.(Asn648Tyr) variant. All the patients carrying *OGT* variants suffer from decreased intellectual ability with IQ scores well below 70. In addition, all patients show mental and physical developmental delay, including intrauterine growth retardation, low birth weight, short stature, drooling, and delayed and/or very restricted language skills.

Brain anomalies were a commonly observed clinical observation among XLID patients with an aberrant *OGT* (Table 1). Three probands with different *OGT* variants present with microcephaly, while megacisterna magna was found in two patients. Additional anatomical brain abnormalities reported among OGT-XLID patients include thin corpus callosum, periventricular leukomalacia, brain atrophy, and cerebral palsy spastic diplegia.

Patients with OGT-XLID variants show a distinct physical dysmorphology (summarised in Table 1 and Fig. 2) suggestive of a syndromic form of XLID. Clinodactyly of the fifth finger was found in five cases, while syndactyly and cone-shaped epiphyses of T2–T5 were reported in one patient (Table 1). Fingers are noted as long and thin in a subset of patients. In addition, some patients have frontal hair upsweeps and high-arched palates. Dysmorphic facial features identified in probands with OGT-XLID variants include triangular, dolichocephalic head with broad and high forehead, full lips, broad nasal root, low set and big ears, full or long philtrum, and hypertelorism (Fig. 2). In patients with detailed clinical description, coarse facial features with drooling are often described, which resembles storage disorders.

Hypotonia, epilepsy/seizures, and genital defects were observed among many of the patients carrying OGT-XLID variants. Six patients were reported to have behavioural problems and two patients presented with sleep abnormalities. Furthermore, some patients display visual and hearing impairment, and suffer from recurrent otitis. Importantly, long fingers and eye abnormalities, including myopia and astigmatism, were observed in a high proportion of patients with OGT-XLID variants, while these characteristics are not common in XLID syndromes. Patients are negative for glycosylated transferrin test results, excluding most N-linked congenital disorders of glycosylation (CDGs).

In conclusion, several clinical features, namely brain abnormalities, general developmental delay, clinodactyly and long fingers, eye abnormalities, and coarse facial features with high forehead and triangular face are shared among the majority of patients with OGT-XLID variants. Based on these common symptoms, we propose that this constitutes a novel syndrome.

### Potential mechanism(s) of disease

#### Hypo O-GlcNAcylation

OGT and O-GlcNAcylated proteins are present in both post- and presynaptic terminals, and O-GlcNAc modified proteins account for 40% of all neuronal proteins and 19% of synaptosome proteins [48]. Perhaps the most obvious hypothesis is that the OGT-XLID variants possess reduced catalytic activity affecting neurodevelopmental pathways (Fig. 2). However, several of the recombinant OGT variants do demonstrate catalytic activity towards both peptide and protein substrates in vitro [29–32] (Table 2). Subsequent analyses in patient-derived or CRISPR/Cas9 engineered cell lines revealed only minor reductions, if any, in global O-GlcNAc levels, with the exception of the c.1942A>T p.(Asn648Tyr) OGT variant that displayed a significant reduction in modified proteins in mESC [33] (Table 2). However, the methods used, primarily western blotting of 1-D SDS-PAGE gels with various pan-O-GlcNAc antibodies, are known to have drawbacks including limited resolution. A much more sensitive approach would be to use combined enrichment and tandem mass tag spectrometry approaches to define the O-GlcNAcome [49–53] and quantitatively compare wild-type and OGT-XLID mutant cell lines and/or tissues. Therefore, while not currently supported by existing findings, this hypothesis for OGT variants’ catalytic activities being impaired for specific substrates cannot be formally excluded.

**Table 2:** Biochemical characteristics of OGT variants found in XLID patients.

OGT variant	c.762G>C p.(Leu254Phe)	c.775G>A p.(Ala259Thr)	c.851G>C p.(Arg284Pro)	c.955G>A p.(Ala319Thr)	c.1016A>G p.(Glu339Gly)	c.1942A>T p.(Asn648Tyr)	c.1701/T>A p.(Asn567Lys)	Total
	TPR domain variants					Catalytic domain variants		
Reference	Vaidyanathan et al. [30] ▲ Gundogdu et al. [59] □ Selvan et al. [31] ●	Selvan et al. [31]	Willems et al. [29] * Selvan et al. [31] ●	Selvan et al. [31]	Selvan et al. [31]	Pravata et al. [32]	Pravata et al. [33]	
Decreased OGT stability	Yes^▲,□,●^	Yes	Yes*	Yes	Yes	No	Yes	**6/7**
Decreased OGT activity	No^▲,●^, yes^□^	No	Yes*, no^●^	No	No	Yes	Yes	2–3/7
Decreased OGT level	Yes^▲^, no^●^	No	Yes*, no^●^	NA	No	No	No	0–2/6
Decreased OGA level	Yes^▲^, no^●^	No	Yes*, no^●^	NA	No	Yes	Yes	**2–4/6**
Decreased O-GlcNAcylation level	No^▲,●^	No	No*^,●^	NA	No	Yes	No	1–2/6
Affected HCF1 processing	No^▲,●^	No	Yes*^,●^	No	No	NA	Yes	2/6

NA indicates cases where no information was available. Publications which provided biochemical analysis of the individual variants are listed in the second row. Symbols indicate the publication in which observations were reported if multiple references are available. Frequently observed phenotypes are highlighted in bold.

#### OGA levels

With the only exception of human embryonic stem cells [31], cell lines carrying the OGT-XLID variants appeared to have significantly reduced levels of OGA at the protein [29, 30, 32, 33] and mRNA [30, 32] level (Fig. 1 and Table 2). Thus, perhaps OGT, OGA, and O-GlcNAc levels operate in transcriptional and/or post-translational feedback mechanisms to maintain O-GlcNAc homoeostasis. Indeed, it has been shown that OGA is capable of upregulating *OGT* gene expression through activation of the transcription factor CCAAT/enhancer-binding protein β (C/EBP-β) [54]. Moreover, inhibition of OGA has been demonstrated to increase *OGA* gene expression, showing that increased O-GlcNAcylation promotes the transcription of *OGA* itself in different cell lines [54]. Thus, variants in *OGT* may lead to reduction of OGA protein/transcript to maintain O-GlcNAc homoeostasis. This also opens the possibility that the OGT-XLID phenotypes are caused by reduced expression of a functional OGA. Indeed, OGA has recently been implicated in neurodevelopmental disease. A recent genome-wide association meta-analysis identified *OGA* intronic variants as linked to nervous system development and, more broadly, to intelligence [55]. Furthermore, knocking down *OGA* in mouse brain leads to microcephaly, hypotonia, and developmental delay [56], suggesting a possible link between OGT-XLID variants and perturbations of OGA levels.

#### HCF1 misprocessing

The second activity of OGT is the promotion of the cleavage of HCF1 [42] (Fig. 1). HCF1 is potent regulator of embryonic neural development and has also been identified as an XLID gene [57]. Variants of *HCFC1* lead to various neurological phenotypes, which include ID. Thus, a possible hypothesis behind the OGT-related XLID phenotypes is that HCF1 is misprocessed. To explore this link, HCF1 processing activity of OGT and the XLID variants has been explored in vitro using recombinant mutant enzymes (Table 2). Changes in HCF1 processing were observed for the c.851G>C p.(Arg284Pro) and c.1701=/T>A p.(Asn567Lys) variants [29, 32]. For the c.1701=/T>A p.(Asn567Lys) variant, which shows the largest effects in vitro, subsequent analysis of HCF1 processing has been carried out in mESC. This approach revealed a direct link between an OGT-XLID variant and misprocessing of HCF1 in cultured cells. Furthermore, RT-qPCR analysis of *GABPA*, a known target of HCF1 and known to mediate synapse-specific gene expression [58], showed increased levels of the gene [32].

Taken together, these results suggest that there may be an association between OGT-XLID variants and HCF1 processing. However, while c.1701=/T>A p.(Asn567Lys) appears to affect HCF1 processing, current data across all variants suggest that HCF1 misprocessing is unlikely to be the general mechanism by which OGT variants lead to the observed XLID phenotypes (Table 2).

#### OGT misfolding

Missense variants in many genes lead to protein misfolding and aggregation, which is particularly toxic to terminally differentiated neurons that entirely rely on proteolytic processing to resolve such aggregates. Therefore another potential mechanism underlying the OGT-related XLID phenotype is that the OGT-XLID variants are unstable and/or aggregate. In support of this hypothesis, detailed crystallographic structural analysis of the c.762G>C p.(Leu254Phe) variant revealed that the larger phenylalanine does not fit the space occupied by the smaller leucine in the wild-type structure, leading to dramatic conformational shifts of up to 15 Å [59]. Atomistic molecular dynamics simulations demonstrated that the amino acid change destabilises the interface between two TPR repeats in the N-terminal domain, increasing the conformational space accessible to OGT. Similarly, the c.1942A>T p.(Asn648Tyr) crystal structure reveals that variant of the conserved asparagine to a tyrosine creates an additional pi–pi stacking interaction within the globular CD. This interaction amplifies the inherent flexibility of the surface exposed loop connecting the two interacting tyrosine residues, which is otherwise rigid [33]. Furthermore, in vitro determination of protein unfolding melting curves demonstrated that all the XLID variants but the c.1942A>T p.(Asn648Tyr) destabilised OGT (Table 2). Collectively, these data show that OGT-XLID variants are destabilised. However, western blotting analysis of OGT protein levels in the majority of cell lines carrying the XLID variants showed unaltered OGT protein levels (Table 2). In summary, despite reductions in the thermal stability of OGT-XLID mutants and some structural evidence for misfolding, there is no evidence yet of formation of toxic aggregates in cultured mammalian cells.

#### OGT interactome

A fifth hypothesis arises from the localisation of several OGT-XLID variants to the TPR domain of OGT. This builds on the prevalent model in the field to reconcile that there are thousands of substrates but only one OGT and thus, OGT is proposed to be regulated by protein interactors that target it to substrates. Since the TPR domain is known to be essential for selection of substrates and their glycosylation [60], it is possible that the TPR XLID variants of OGT lead to impaired recognition and binding to substrates, likely in a tissue-specific manner. Single-amino acid substitutions in TPRs have been documented to interrupt highly specific protein–protein interactions [61]; therefore, it is possible that TPR variants in OGT interrupt specific substrate and/or adaptor protein interactions. The loss of interaction with adaptor proteins and/or glycosylation of the target substrate could lead to functional consequences for the substrate protein that have yet to be defined. However, the presence of CD variants in OGT that lead to XLID with similar phenotypes complicate this hypothesis. In order to address this, the OGT interactome in appropriate tissues and/or differentiated cell lines must first be defined. Comparisons can then be made between the wild-type and XLID variant OGT interactomes to identify differential interactors and evaluate impact on a subset of adaptor proteins and their binding partners that could easily be overlooked via western blotting of whole cell extracts. Defining the O-GlcNAcome and O-GlcNAc cycling rates, will be essential to evaluate the role of catalytic OGT variants in this hypothesis.

## Discussion

Pathogenic variants in OGT are mainly associated with intellectual and developmental disability, microcephaly, eye abormalities, and coarse facial features with high, broad forehead, and triangular face. This might be an X-linked CDG like ALG13-CDG, ATP6AP1-CDG, ATP6AP2-CDG, MAGT1-CDG, PIGA-CDG, SLC35A2-CDG, SSR4-CDG, and TRAPPC2-CDG [62]. Therefore, we term this syndrome OGT-CDG.

This review has discussed a number of hypotheses, some of which have been partially tested, as to the biological mechanisms underpinning the patient phenotypes (Fig. 3). The hypotheses of decreased OGT activity as a glycosyltransferase or protease are not supported as a common mechanism by currently existing data on multiple variants. The contribution of decreased OGT/OGA levels due to decreased OGT stability, the possibility for aberrant interactions between OGT-CDG variants and target substrates, and alterations in O-GlcNAc cycling rates are compelling hypotheses that still need to be explored. To achieve this goal, new approaches to examining the OGT interactome, the O-GlcNAcome, and the dynamics of the O-GlcNAc modification must be developed. Generation and characterisation of CRISPR/Cas9 engineered stem cells and model organisms will significantly contribute to the elucidating of the biological processes that underlie the OGT-CDG pathology. First attempts have been made with the generation of human and mouse ES cells and a fly model of the c.1701=/T>A p.(Asn567Lys) variant, although further work is required including vertebrate models of the disorder. Indeed, such disease models and in-depth the understanding of the disease mechanism they offer will be instrumental in devising a potential therapeutic intervention strategy.

Many studies have reported dietary interventions with monosaccharides such as galactose, fucose, or mannose as successful therapies used in patients suffering from CDGs [63]. These are aimed at increasing intracellular concentrations of metabolites specific for each subtype and, interestingly, were successfully used in CAD-CDG with uridine supplementation [64], SLC39A8-CDG with manganese [65], and TMEM165-CDG with galactose [66]. A similar approach could be considered for OGT variants. Indeed, it has been observed that supplementation of cell culture media with glucosamine (GlcN), bypassing the feedback inhibition of the enzyme GFAT, leads to elevated levels of UDP-GlcNAc, which are known to regulate OGT activity [18, 67]. For the patient with the c.1942A>T p.(Asn648Tyr) variant, GlcN supplementation is being explored with some positive effects [33]. Another interesting approach could be the use of OGA inhibitors. Indeed, many OGA inhibitors have been described and showed to be cell permeable [68–70], suggesting this may be a possible future therapeutic approach.

A suitable diagnostic test has to be devised to detect OGT-XLID in patients. A commonly used CDG diagnostic test is isoelectrofocusing of serum transferrin. Since transferrin is only N-glycosylated, it detects only N-glycosylation disorders associated with sialic acid deficiency and thus not OGT-CDG. Since patient-derived cells show reduced OGA, we propose western blotting for OGA protein levels as a rapid initial diagnostic test for OGT-CDG.

In conclusion, more than 30 years after the discovery of the O-GlcNAc modification and 20 years after the cloning of OGT [17, 18], the O-GlcNAc system has now been linked directly to a congenital disorder and we can take advantage of the huge strides made in understanding this modification in other settings [71] in order to uncover its role in this syndromic form of OGT-CDG.
